# The Interaction of Dipole Modifiers with Polyene-Sterol Complexes

**DOI:** 10.1371/journal.pone.0045135

**Published:** 2012-09-21

**Authors:** Olga S. Ostroumova, Svetlana S. Efimova, Evgeny G. Chulkov, Ludmila V. Schagina

**Affiliations:** Institute of Cytology of the Russian Academy of Sciences, St. Petersburg, Russia; Nagoya University, Japan

## Abstract

Recently, we showed that the effect of dipole modifiers (flavonoids and styrylpyridinium dyes) on the conductance of single amphotericin B (AmB) channels in sterol-containing lipid bilayers primarily resulted from changes in the membrane dipole potential. The present study examines the effect of dipole modifiers on the AmB multi-channel activity. The addition of phloretin to cholesterol-containing membranes leads to a significant increase in the steady-state AmB-induced transmembrane current. Quercetin significantly decreases and RH 421 increases the current through ergosterol-containing bilayers. Other tested flavonoids and styrylpyridinium dyes do not affect the channel-forming activity of AmB independently on the sterol composition of the bilayers. The effects obtained in these trials may instead be attributed to the direct interaction of dipole modifiers with AmB/sterol complexes and not to the effect of dipole potential changes. The presence of double bonds in the Δ7 and Δ22 positions of sterol molecules, the number of conjugated double bonds and amino sugar residues in polyene molecules, and the conformation and adsorption plane of dipole modifiers are important factors impacting this interaction.

## Introduction

**Figure 1:**
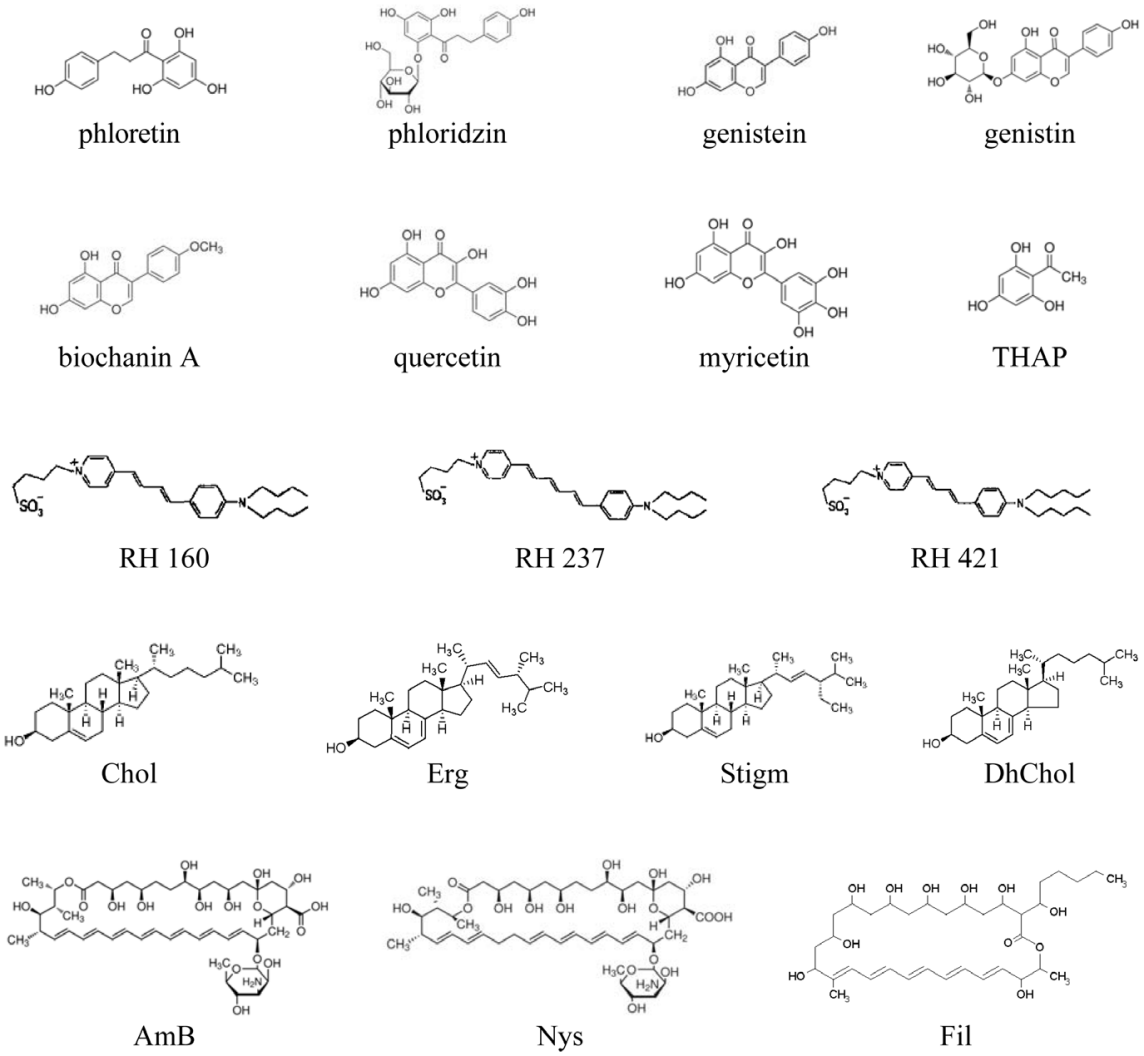
The chemical structures of the flavonoids, styryl dyes, sterols and polyenes.

In recent years, there has been growing interest in understanding the role that lipid bilayer properties have on the channel-forming action of various membrane-active biomolecules. One of the factors that determine the interaction between the membrane and certain compounds, such as pore-forming toxins and antimicrobial agents, is the membrane dipole potential, which originates from the specific orientation of the water and lipid dipoles at the membrane’s interface [1]–[11]. This dipole potential can be varied by membrane-active agents known as dipole modifiers. Among these dipole modifiers are flavonoids, such as phloretin and phloridzin, which are able to reduce the membrane dipole potential [12]. Flavonoids are widely distributed in higher plants and play an essential role in their metabolism. Flavonoids exhibit anti-inflammatory, anti-allergic, antioxidant, antiviral and antitumor activity [13], [14]. As flavonoids evince low toxicity, high biological activity, and the ability in certain cases to modify membrane properties, they are excellent prospects for use as regulators of the toxicity of different pore-forming toxins and the therapeutic activity of antimicrobial compounds. It is also well known that certain sterols, cholesterol, 6-ketoholestanol and styryl RH dyes (Fig. 1) increase the dipole potential of membranes [15], [16]. As sterols are constitutive components of the plasma membrane, and their presence defines the specificity of action for many pharmacological agents, the study of the channel-forming activity of toxins and antimicrobial compounds in the lipid bilayers of different sterol composition is important for predicting the effects of these compounds on different cell types.

In the literature, recent evidence has indicated that the effect of dipole modifiers on channel formation in the membranes is mediated not only by the changes in the membrane dipole potential but also by methods such as the control of membrane heterogeneity and spontaneous curvature by dipole modifiers, as well as the direct interaction of dipole modifiers with the channel-forming molecules [9], [17]–[19]. These data indicate the need to consider these specific effects when addressing the mechanisms of the influence of dipole modifiers on the channel-forming activity of different compounds.

Membrane-active polyene macrolides are clinically important antifungal agents. Because of their broad spectrum of action against fungi and other prokaryotic microbes, certain of these compounds are widely used for medicinal purposes [20]–[22]. Their chemical structures feature a large ring containing multiple conjugated double bonds on one side and multiple hydroxyl groups bonded to the other side of the ring [23]. Polyenes can most simply be classified by their number of conjugated double bonds. Nystatin and amphotericin are the most studied polyene antimycotics and are related to tetranes and heptanes, respectively. Both molecules are characterized by having 38 atoms in their macrolide rings and the presence of a bonded *d*-mycosamine group. Filipin is related to a pentane and is the simplest of the polyene antibiotics without an amino sugar residue, with a 28-atom macrolide ring. The biological activity of polyenes may be related to pore formation in the membranes of target cells [24]–[27]. According to the “primary complex” hypothesis, polyenes associate with sterols in the membrane to form binary complexes, which assemble into barrel-stave channels [28]. Polyenes are poorly selective between fungal and mammalian cells and thus are highly toxic [29]–[31]. To rationally design less toxic preparations, the mechanism of action of polyenes and their interaction with potential activators and inhibitors at the molecular level must be elucidated.

In the membrane, the negatively charged carboxyl group (COO^−^) of AmB is slightly shifted toward the aqueous phase compared with the protonated amino group (NH_3_^+^) [32]. One might think that the decrease in the membrane dipole potential, i.e., the electric field (which is positive inside the hydrocarbon core), would tend to promote the orientation of the polyene polar head needed for pore formation that was described above and therefore enhance polyene activity. The opposite may hypothesized for the case of increasing dipole potential. These assumptions led us to investigate the effect of dipole modifiers on the channel-forming activity of polyenes. Through this research, we revealed that the stability of the polyene/sterol complexes is responsible for the multichannel polyene activity. The influence of different structural features of polyene, sterol and dipole modifier molecules on complex stability is discussed.

## Materials and Methods

All chemicals were of reagent grade. Synthetic 1,2-diphytanoyl-*sn*-glycero-3-phosphocholine (PC), cholesterol (Chol), ergosterol (Erg), stigmasterol (Stigm) and 7- dehydrocholesterol (DhChol) were obtained from Avanti Polar Lipids, Inc. (Pelham, AL). Phloretin (3-(4-hydroxyphenyl)-1-(2,4,6-trihydroxyphenyl)-1-propanone), phloridzin (1-[2-(β-D-Glucopyranosyloxy)-4,6-dihydroxyphenyl]-3-(4-hydroxyphenyl)-1-propanone), genistein (5,7-Dihydroxy-3-(4-hydroxyphenyl)-4H-1-benzopyran-4-one), genistin (Genistein-7-O-β-D-glucopyranoside), 2′,4′,6′-trihydroxy-acetophenone monohydrate (THAP), quercetin (2-(3,4-Dihydroxyphenyl)-3,5,7-trihydroxy-4H-1-benzopyran-4-one), myricetin (3,3′,4′,5,5′,7-Hexahydroxyflavone), and biochanin A (5,7-Dihydroxy-4′-methoxyisoflavone) were purchased from Sigma Chemical (St. Louis, MO), whereas RH 421 (N-(4-sulfobutyl)-4-(4-(4-(dipentylamino)phenyl)butadienyl) pyridinium, inner salt), RH 237 (*N*-(4-sulfobutyl)-4-(6-(4-(dibutylamino)phenyl)hexatrienyl)pyridinium, inner salt), and RH 160 (*N*-(4-sulfobutyl)-4-(4-(4-(dibutylamino)phenyl)butadienyl)pyridinium, inner salt) were purchased from Molecular Probes (Eugene, OR). The water used in this study was double distilled and deionized. The 2 M KCl solutions used were buffered with 5 mM HEPES, pH 7.0. Amphotericin B (AmB), nystatin (Nys) and filipin III (Fil) were purchased from Sigma Chemical (St. Louis, MO). The structures of all the flavonoids, RH dyes, sterols, and polyenes are shown in Fig. 1.

Planar lipid bilayers that were virtually solvent-free were formed using a monolayer-opposition technique [33] on a 50-µm-diameter aperture in the 10-μm-thick Teflon film separating the two (*cis* and *trans*) compartments of the Teflon chamber. The aperture was pretreated with hexadecane. Lipid bilayers were made from 67 mol% PC and 33 mol% sterol (Chol, Erg, Stigm or DhChol). After the membrane was completely formed and stabilized, AmB from stock solution (0.1 mg/ml in DMSO), Nys from stock solution (1 mg/ml in DMSO) or Fil from stock solution (0.1 mg/ml in ethanol) were added to both compartments to a final concentration that ranged from 10^−8^ to 10^−6^ M. Ag/AgCl electrodes with agarose/2 M KCl bridges were used to apply the transmembrane voltage (*V*) and measure the transmembrane current (*I*). “Positive voltage” refers to the situation in which the *cis*-side compartment is positive with respect to the *trans*-side. All experiments were performed at room temperature.

The two-sided addition of flavonoids (phloretin, phloridzin, genistein, genistin, THAP, quercetin, myricetin or biochanin A) or styryl dyes (RH 421, RH 237 or RH 160) from stock mM solutions in ethanol or DMSO to the membrane-bathing solution yielding final concentrations of 20 µM for the different flavonoids and 5 µM for the various RH dyes was used to modulate the channel-forming activity of AmB. The final concentration of DMSO or ethanol in the chamber did not exceed 0.1%. These concentrations of solvents did not affect the integrity of lipid bilayers and did not increase their conductance.

The current measurements were conducted using an Axopatch 200B amplifier (Axon Instruments) in the voltage clamp mode. The data were digitized with Digidata 1440A and analyzed using pClamp 10 (Axon Instruments) and Origin 7.0 (OriginLab). The current tracks were processed through an 8-pole Bessel 100-kHz filter. The channel-forming activity of polyenes (AmB, Nys or Fil) in the absence and after the introduction of the modifier (flavonoids or styryl dyes) was characterized by a steady-state transmembrane current (*I_∞_*) under the given experimental conditions (*V* = 50 mV and the given polyene concentration). Mean ratios (*I_∞_/I_∞_^0^*) of steady-state transmembrane current induced by AmB in the presence (*I_∞_*) and in the absence of modifiers (*I_∞_^0^*) were averaged from 3 to 9 bilayers (mean ± se).

The changes in K^+^-nonactin steady-state conductance were measured to estimate the changes of the dipole potential of Cho-l and Erg- containing membranes after AmB addition into a bilayer bathing solution up to 10^−9^ M (0.1 M KCl, 5 mM Hepes, 7.4). The corresponding calculations were performed under the assumption that the membrane conductance is related to the bilayer dipole potential by the Boltzmann distribution [12]: 
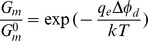
, where *G_m_* and *G_m_^0^* are the steady-state membrane conductance induced by K^+^-nonactin in the presence and the absence of AmB, respectively, Δ*φ_d_* represents the changes of the membrane dipole potential after the addition of AmB into a bilayer bathing solution, and *q_e_*, *k*, and *T* have their typical meanings.

## Results and Discussion

**Figure 2:**
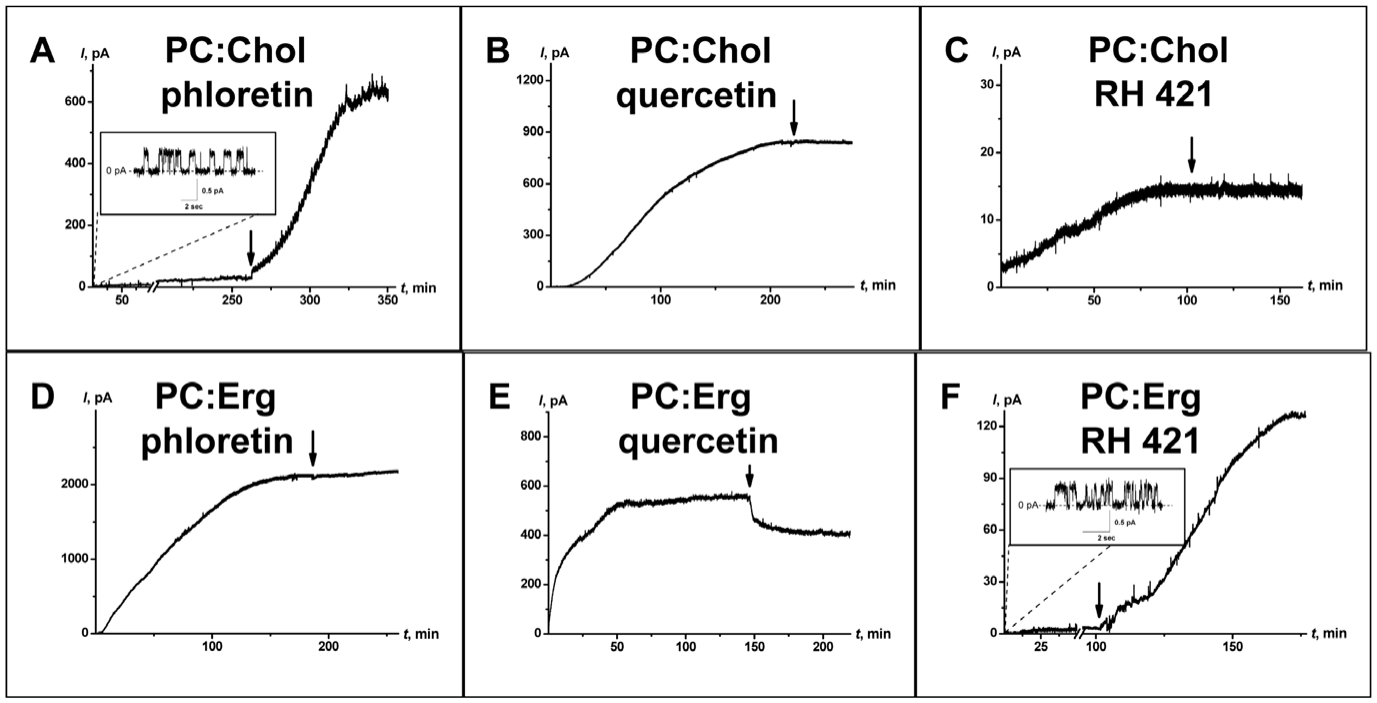
The effect of phloretin, quercetin, and RH 421 on the steady-state transmembrane current induced by AmB. The membranes were composed of PC:Chol (67∶33 mol%) (upper panel) or PC:Erg (67∶33 mol%) (lower panel) and bathed in 2.0 M KCl, pH 7.0. The addition of 20 µM phloretin (A, D), 20 µM quercetin (B, E) or 5 µM RH 421 (C, F) to the bilayer bathing solution is indicated by an arrow. *V* = 50 mV.

Figure 2 illustrates the time course of the AmB-induced transmembrane currents obtained after the addition of the dipole modifiers of 20 µM phloretin, 20 µM quercetin, or 5 µM RH 421 to both sides of lipid bilayers containing 33 mol% of either Chol (upper panel) or Erg (lower panel). One can observe that the effects of dipole modifiers are dependent on the type of sterol in the bilayer. In particular, it can be noted that: (i) phloretin significantly increases the steady-state current (*I_∞_*) through Chol-containing bilayers, but it has no effect on Erg-containing membranes; (ii) quercetin does not affect *I_∞_* in Chol-containing membranes, but it decreases *I_∞_* in Erg-containing bilayers; and (iii) RH 421 has no effect on *I_∞_* in Chol-containing membranes, whereas it significantly increases the steady-state current through Erg-containing bilayers. As addition of phloretin or quercetin leads to significant dipole potential reduction, whereas introduction of RH 421 causes growth of the dipole potential in both Chol- and Erg- containing bilayers [34], [35], the observed effects of dipole modifiers on the channel-forming activity of AmB cannot be attributed to changes in the membrane dipole potential.

The independence of *I_∞_* from the changes in bilayer dipole potential supports the notion that dipole-dipole interactions provide only small contributions to the channel-forming activity of AmB. This can be explained by the small value of the projection of the dipole moment of AmB molecule in the direction that is normal to the plane of the membrane. In this case, the contribution of the above orientation of the AmB dipoles to the increase in the bilayer dipole potential should be negligible. This forces us to measure the changes of the dipole potential of Chol- and Erg-containing bilayers (Δ*φ_d_*) after the addition of AmB (see Materials and Methods), whereupon we find that Δ*φ_d_* is equal to 0.2±0.1 mV.

The observed effects of dipole modifiers on the channel-forming activity of AmB may proceed instead from the direct interaction of these modifiers with the AmB-sterol complexes that form the pores. Recently, we demonstrated that the conductance of single AmB channels depends on the membrane dipole potential [1]. However, RH 421 is more effective in Erg-containing bilayers than in Chol-containing membranes. By contrast, quercetin does not affect the AmB-channel conductance in Erg-containing membranes but does impact the conductance of Chol-containing bilayers. Despite these differences, these modifiers alter the magnitude of the dipole potential of Chol as well as Erg-containing membranes [34], [35]. Thus, direct interactions of RH 421 and quercetin with AmB-sterol complexes must contribute to both the single and multichannel activities of AmB.

We asked what bonds and functional groups in the molecules of dipole modifiers, sterols and polyene antibiotics are involved in these interactions. Table 1 presents the mean ratio (*I_∞_/I_∞_^0^*) of the steady-state transmembrane current induced by AmB in the presence (*I_∞_*) and absence (*I_∞_^0^*) of different flavonoids in Chol- and Erg-containing bilayers. For the structural features of different flavonoids, please see Fig. 1. We tested chalcones (phloretin and its glycoside phloridzin, in particular, to decrease hydrophobicity and increase molecule size); flavones (biochanin A, genistein, and genistin, in particular, to decrease hydrophobicity); flavonols (quercetin and myricetin, in particular, to decrease hydrophobicity); and a flavonoid analog of smaller size – phloglucinol (THAP). It should be noted that the relative mobility of the two benzene rings in the molecules of chalcones (particularly phloretin) allows them to take on a bilayer conformation similar to a “hairpin” [36]. In the case of flavones and glycones (biochanin A and genistein) and flavon-3-ols (quercetin and myrecetin), the only possible conformation is rod-shaped, with a long axis of the molecule that is perpendicular to the membrane surface. From Table 1, one can see that phloretin increases the steady-state transmembrane current induced by AmB in Chol-containing bilayers, whereas quercetin decreases *I_∞_* in Erg-containing membranes. Other tested flavonoids have no effect on the channel-forming activity of AmB independently of the sterol composition of the bilayers. Presumably, in the case of phloretin, the “hairpin” conformation and the smaller size of its aglycone are important features of its effect. As myricetin does not influence AmB activity in Erg-containing bilayers, an OH-group in the 3-position of the flavonol molecule is irrelevant to the interaction of quercetin with the AmB/Erg complex. According to Tarahovsky [36], the distribution of quercetin in the bilayer is biphasic: quercetin molecules should be localized at the boundary between the polar and hydrophobic regions and within the hydrophobic region of the bilayer. The latter fraction of quercetin may be responsible for its interaction with AmB-Erg complexes.

**Table 1:** The mean ratios (*I_∞_/I_∞_^0^*) of the steady-state transmembrane currents induced by AmB in the presence (*I_∞_*) and absence (*I_∞_^0^*) of flavonoids.

membrane composition	phloretin	phloridzin	genistein	genistin	biochanin A	quercetin	myricetin	THAP
PC:Chol	11.0±2.9	1.2±0.1	1.1±0.1	0.9±0.2	1.1±0.2	1.1±0.3	1.1±0.1	1.0±0.1
PC:Erg	0.8±0.1	1.0±0.2	0.8±0.1	0.8±0.2	0.9±0.1	0.5±0.1	0.9±0.1	1.1±0.1

The bilayers were composed of PC:Chol (67∶33 mol%) or PC:Erg (67∶33 mol%) and bathed in 2.0 M KCl, pH 7.0.

Table 2 presents the mean ratio of steady-state transmembrane current induced by AmB in the presence and absence of different RH dyes in Chol- and Erg-containing bilayers. These dyes differ from each other in the lengths of their “tails” and the polyene fragment present between the rings (Fig. 1). One can observe that only RH 421 has a major effect on Erg-containing bilayers. According to Passechnik and Sokolov [37], RH 421 has a depth of chromophore dipping in the bilayer that is less than that of RH 160 but greater than that of RH 237. It thus appears most probable that the localization of the chromophore in the bilayer and the orientation of the molecule of RH 421 in the membrane in a direction closer to normal (because of the long hydrocarbon tails) play key roles in its interaction with the AmB/Erg complex.

**Table 2:** The mean ratios (*I_∞_/I_∞_^0^*) of the steady-state transmembrane currents induced by AmB in the presence (*I_∞_*) and absence (*I_∞_^0^*) of styryl dyes.

membrane composition	RH 421	RH 237	RH 160
PC:Chol	1.1±0.1	0.8±0.2	1.2±0.2
PC:Erg	15.2±6.1	0.9±0.1	1.1±0.2

The bilayers were composed of PC:Chol (67∶33 mol%) or PC:Erg (67∶33 mol%) and bathed in 2.0 M KCl, pH 7.0.

The presence of hydroxyl groups and unsaturated bonds of dipole modifiers may suggest a contribution of these molecules to a network of hydrogen bonds and π-π electronic interactions between the sterol and AmB molecules stabilizing AmB/sterol-complexes. Neumann et al. [28] demonstrated that AmB/Chol and AmB/Erg complexes had different molecular geometries. The structure of the AmB/Chol-complex prevents sterols from establishing close contact and forming a hydrogen bond between the OH-group at C-43 of the AmB amino sugar residue and the 3β-OH group of the sterol molecule, instead causing the sterol to move deeper into the membrane hydrocarbon core relative to Erg. Due to their rigid form and parallel orientation, Erg and AmB in the AmB/Erg complex are held together by stronger van der Waals forces than are observed between Chol and AmB in the AmB/Chol complex. This attractive effect maintains the Erg OH-group closer to the AmB polar head than the corresponding group of Chol. We speculate that phloretin can play the role of a mediator for the formation of hydrogen bonds between the OH-group of Chol and the AmB polar head, which stabilizes the complex by leading to the formation of additional mediated point-to-point interactions that maximize van der Waals forces. In the case of the AmB/Erg complex of coplanar and parallel molecules, RH 421 may participate in π-π interactions (between the polyene chromophore of AmB, the sterol rings and the side chain of the sterol) due to its polyene fragment between two phenol rings and may also insert its polar groups into a hydrogen bond network (including the polar groups of an antibiotic and the OH-group of a sterol). In this case, the third constituent may contribute to the orienting force needed for complex formation. The reduction of channel forming activity in the presence of quercetin suggests that this agent destabilizes AmB/Erg-complex by interacting with it inside the hydrophobic region of the bilayer.

Erg differs structurally from Chol in that Erg has two more double bonds (Δ7 and Δ22) and an additional 24β-methyl group (Fig. 1). Stigm has a core structure identical to that of Chol and a C-17 side chain structure similar to that of Erg (including a Δ22-double bond and a 24β-ethyl group). DhChol has a core structure identical to that of Erg (including a Δ7-double bond) and a C-17 side saturated chain structure identical to that of Chol. Table 3 presents the results from measuring the mean ratios of steady-state transmembrane currents induced by AmB in the presence of phloretin or RH 421 and in the absence of any modifiers in Stigm- and DhChol-containing bilayers. Phloretin increases AmB activity in these two bilayers, whereas RH 421 does not impact AmB activity in these cases in any significant way. This result implies that the geometry of the AmB/Stigm and AmB/DhChol complexes more closely approximate the geometry of the AmB/Chol complex than that of the AmB/Erg complex. Most likely, the absence of one double bond (Δ22 or Δ7) prevents the sterol from establishing close contact with the amino sugar residue of AmB. These data contradict the results from molecular modeling of AmB/sterol complexes in a study by Baran et al. [38], which indicated that only the Δ22-double bond plays a key role as a point-to-point interaction that promotes maximization of the van der Waals forces between the polyene and sterol molecules and thereby stabilizes their complex.

**Table 3:** The mean ratios (*I_∞_/I_∞_^0^*) of steady-state transmembrane currents induced by AmB in the presence (*I_∞_*) and absence (*I_∞_^0^*) of modifiers in bilayers composed of PC:Stigm (67∶33 mol%) and PC:DhChol (67∶33 mol%).

membrane composition	phloretin	RH 421
PC:Stigm	5.3±3.1	1.1±0.1
PC:DhChol	1.7±0.3	1.2±0.1

The membranes were bathed in 2.0 M KCl, pH 7.0.

Our next step was to vary the third participant of this interaction, namely, the polyene antibiotic. For this purpose, we chose two compounds to test. First, we used the closest analogue of AmB, nystatin, which differs from AmB by the absence of a double bond in the polyene chain and thus should produce distinct π-π electronic interactions in the ternary complex. We also tested filipin, a polyene without an amino sugar in the polar “head”, as this structural difference should change the polyene contribution to a network of hydrogen bonds in the hydrophilic region of the bilayer.

Table 4 presents the mean ratios of steady-state transmembrane currents induced by Nys and Fil in the presence and absence of different dipole modifiers in PC:Chol and PC:Erg bilayers after the addition of 20 µM phloretin, 20 µM quercetin or 5 µM RH 421. In both lipid conditions, Nys qualitatively demonstrates current traces similar to those induced by AmB in Chol-containing bilayers. These data suggest that the Nys/Erg complex is less favorable than the AmB/Erg complex. Thus, the absence of a single double bond in the polyene molecule, which leads to the break in the sequence of conjugated double bonds and changes in the π-π interactions between the polyene chromophore of AmB, the sterol and RH 421, prevents Nys from its closest contact with the sterol. The interaction of dipole modifiers with the Fil/sterol complex does not depend on the sterol type. This fact may reflect the similar geometry of Fil/Chol and Fil/Erg complexes. The absence of the amino sugar residue in the filipin “head” prevents the formation of the hydrogen bond between the OH-group at C-43 of an amino sugar residue and the 3β-OH group of the sterol molecule, which occurs in the case of the AmB/Erg complex. This lack may decrease van der Waals forces and make the Fil/Chol and Fil/Erg complexes less favorable than the AmB/Erg complex. Moreover, Fil molecules, due to their higher hydrophobicity, should be localized more deeply inside the hydrophobic region of the bilayer than are the AmB and Nys molecules that may be responsible for facilitating the interaction of Fil/sterol complexes with quercetin fractions in the hydrophobic region and thereby enhancing the channel-forming activity of Fil.

**Table 4:** The mean ratios (*I_∞_/I_∞_^0^*) of steady-state transmembrane currents induced by different polyenes in the presence (*I_∞_*) and absence (*I_∞_^0^*) of modifiers in PC:Chol (67∶33 mol%) and PC:Erg (67∶33 mol%) bilayers bathed in 2.0 M KCl, pH 7.0.

polyenes	membranecomposition	phloretin	quercetin	RH 421
Nys	PC:Chol	5.1±1.4	1.0±0.1	1.1±0.1
	PC:Erg	3.8±0.7	1.0±0.1	1.0±0.1
Fil	PC:Chol	2.2±0.7	11.0±4.1	1.0±0.1
	PC:Erg	2.8±0.8	10.4±3.1	1.1±0.1

The geometry and stability of polyene/sterol complexes should not be treated as a single key factor affecting polyene channel formation and therefore impacting antifungal activity. For instance, the presence of the double bond at the C-22 position in the terminal hydrocarbon chain of Erg and Stigm should make the structures of these compounds more rigid than those of Chol and DhChol. Stronger van der Waals forces between the rigid molecules (AmB and either Erg or Stigm) stabilize the corresponding complexes. For Nys, a hydrogenation of the double bond in the polyene chain (compared with AmB) causes slower channel formation than is observed for AmB [39]. This discrepancy appears to be due to the greater flexibility of the molecule at the point where the double bonds break, which makes it more difficult for the molecule to form a complex with sterol. Based on such reasoning, one may hypothesize that AmB should be more active in Erg- and Stigm-containing membranes compared with Chol- and DhChol-containing membranes. In addition, Nys should be less active than AmB. However, Hsuchen and Feingold [40] demonstrated that the polyene-induced glucose release from dipalmitoyl lecithin liposomes is drastically suppressed by the incorporation of Chol and Stigm but unchanged by the incorporation of Erg and DhChol. Moreover, the AmB- and Nys-induced releases of glucose were similar. Thus, it is clear that the selectivity of AmB is caused not only by the preferential formation of a complex of AmB with Erg over Chol but also may result from differential preorganization of membranes by different sterols. Many authors consider the indirect effect of the sterol on bilayer thickening and ordering to be the primary mechanism for AmB and Nys channel formation instead of direct interactions between sterols and AmB [41]–[43]. In addition, from greatest to least, Erg, Chol, DhChol, and desmosterol (the close analog of Stigm) have been shown to produce different ordering effects on saturated bilayers [44]. These data are also not in agreement with the measurements of polyene-induced glucose release. It should furthermore be noted that lipid packing depends upon both sterol and polyene effects. AmB restricts the molecular motion of the choline fragment in both Erg- and Chol-containing membranes but increases the segmental motional freedom in the hydrophobic core of Erg-containing membranes only [45].

In the literature, recent evidence demonstrates that dipole modifiers may affect the packing of lipids and produce a non-uniform distribution of ordered and disordered lipid phases in the membrane [9], [18], [36]. RH 421 may increase the spontaneous curvature of the monolayer [9] and therefore may contribute to membrane ordering. Tarahovsky et al. [36] discussed the ability of flavonoids to influence lipid packing characteristics. Flavonoid molecules that preferentially localize to the hydrophobic region of the bilayer (such as quercetin) can initiate raft formation, whereas the molecules located in the polar interface region of the bilayer (such as phloretin) can fluidize membranes. Amphotericin B prefers an intermediate membrane phase between the liquid-disordered state and the liquid-ordered state (rafts) [46]. Therefore, the indirect effect of dipole modifiers on phase separation in the membrane should also be considered when assessing the activity of polyenes in sterol-containing bilayers.
